# Comparison of Surgical Outcomes Between Vertebral Body Stenting (VBS) and Balloon Kyphoplasty (BKP)—Multicenter Cohort Study

**DOI:** 10.3390/jcm15093371

**Published:** 2026-04-28

**Authors:** Akiyoshi Miyamoto, Ingrid Ignacio, Masato Tanaka, Shinya Arataki, Tadashi Komatsubara, Ryo Ugawa, Nitin Jaiswal, Pankaj Kumar Sharma, Yoshiaki Oda, Koji Uotani

**Affiliations:** 1 Department of Orthopaedic Surgery, Okayama Rosai Hospital, Okayama 702-8055, Japan; hello.akkun.11.136@icloud.com (A.M.); ingriddignacio@gmail.com (I.I.); araoyc@gmail.com (S.A.); t.komatsubara1982@gmail.com (T.K.); ryougawa0412@yahoo.co.jp (R.U.); dr.jaiswalnitin@gmail.com (N.J.); dr.pankajkristwal@gmail.com (P.K.S.); 2 Department of Orthopaedic Surgery, Okayama University Hospital, Okayama 700-8558, Japan; odaaaaaaamn@yahoo.co.jp (Y.O.); coji.uo@gmail.com (K.U.)

**Keywords:** osteoporotic vertebral fracture, balloon kyphoplasty, vertebral body stenting

## Abstract

**Background/Objectives:** Vertebral body stenting (VBS) and balloon kyphoplasty (BKP) are widely used for the treatment of osteoporotic vertebral fractures (OVFs). However, it remains unclear whether the theoretical biomechanical advantages of VBS translate to superior clinical or radiographic outcomes. This study aimed to compare VBS and BKP with respect to clinical outcomes, radiographic parameters, and complications. **Methods:** In this multicenter retrospective comparative cohort study, 123 patients with OVF treated with VBS (*n* = 24) or BKP (*n* = 99) were analyzed. VBS was indicated for complex fracture patterns, including severe endplate injury, split-type fractures, and absence of interbody sclerosis; other fractures were treated with BKP. Pain outcomes, operative parameters, cement volume and leakage, and radiographic measures of vertebral kyphosis angle (VKA) and local kyphosis angle (LKA) were assessed. For group comparisons, we used independent-samples t tests or Mann–Whitney U tests for continuous variables and chi-squared or Fisher’s exact tests for categorical variables. **Results:** Baseline demographics and bone mineral density were comparable between groups. Surgical time was longer for VBS (39 ± 6 vs. 35 ± 9 min, *p* = 0.007). Both procedures produced significant pain reductions (*p* < 0.001), and postoperative VAS did not differ between VBS and BKP (18 ± 11 vs. 13 ± 12 mm, *p* = 0.06). Although VKA immediately after surgery was lower for VBS (4.8 ± 4.4° vs. 7.0 ± 4.9°, *p* = 0.03), the magnitude of correction, VKA, and LKA at final follow-up were comparable. Cement volume was similar (6.4 ± 1.4 vs. 6.7 ± 1.9 mL, *p* = 0.45), but cement leakage occurred more frequently with VBS (54% vs. 24%, *p* = 0.005). Rates of adjacent vertebral fracture (13% vs. 26%, *p* = 0.12) and revision surgery (4% vs. 8%, *p* = 0.44) were comparable between groups. **Conclusions:** Despite VBS being reserved for more complex fracture morphologies with split-type fractures and severe endplate defects, while BKP was generally used for uncomplicated OVF cases, VBS provided pain relief and radiographic correction comparable to BKP.

## 1. Introduction

With over 13 million people in Japan currently living with osteoporosis, osteoporotic vertebral fractures (OVFs) represent approximately half of all fragility fractures [1], placing a considerable and growing burden on both individual patients and the wider healthcare system [2,3,4]. Beyond Japan, OVF represents a leading cause of disability, reduced quality of life, and increased mortality among older adults, with substantial healthcare costs reported in Europe and worldwide [5]. Many affected individuals experience persistent pain, progressive vertebral collapse, and gradual progression of a kyphotic deformity, necessitating surgical intervention [4,6]. Approximately one third of patients with OVF seek medical attention [3,7] due to severe back pain, which in turn leads to reduced mobility, loss of independence, and progressive functional decline [7,8]. Conservative management—including analgesia, bracing, and physical therapy—is typically employed as first-line treatment; however, surgical intervention is often indicated for patients with intractable pain, neurologic compromise, or radiographic evidence of non-union [6,7,8,9]. In addition, several risk factors for nonunion, including posterior wall injury [10], intravertebral effusion, and high-intensity or diffuse low-intensity areas on T2-weighted MRI [8], have been previously described, and patients exhibiting these features may benefit from surgical intervention [8,10].

Percutaneous vertebral augmentation techniques have been developed to provide rapid pain relief, stabilize the fractured vertebral body, and, in selected cases, restore lost vertebral height [11,12,13,14,15,16]. Balloon kyphoplasty (BKP) involves the insertion and inflation of an inflatable bone tamp within the collapsed vertebral body to create a cavity and partially re-expand the vertebra before injection of cement [13,17]. Multiple studies have demonstrated that BKP is effective for short-term pain reduction, improvement in functional status, and reduction in kyphosis [9,11,12,13,18]. However, an inherent limitation of BKP is the partial loss of restored vertebral height that occurs after balloon deflation, prior to cementation [19]. To address this effect, vertebral body stenting (VBS) was developed, incorporating an expandable scaffold, conceptually similar to vascular stents, that is intended to maintain height restoration after balloon deflation [20,21]. Biomechanical investigations have shown that VBS reduces the “deflation effect,” thereby improving kyphosis correction compared with BKP, thereby supporting the theoretical advantage of the stent-based system [19,21]. However, clinical studies have reported conflicting findings on whether these biomechanical benefits translate into superior radiographic or clinical outcomes [22,23,24,25,26].

Against this background, we hypothesized that, despite being preferentially used for more complex fractures, VBS would achieve pain relief and radiographic correction comparable to BKP, with a lower rate of cement leakage and adverse events. Clarifying the results of these techniques in routine practice is essential to guide procedure selection for patients with osteoporotic vertebral fractures, particularly those with high-risk fracture morphologies.

## 2. Materials and Methods

### 2.1. Research Design

**Figure 1:**
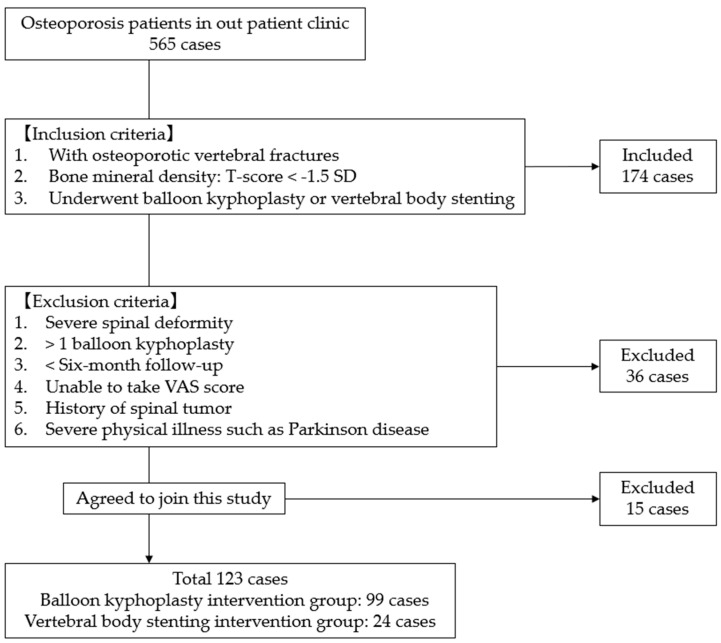
Patient selection flow diagram.

This multicenter retrospective cohort study was conducted at two institutions in Japan (Okayama Rosai Hospital and Miyamoto Hospital). This study was approved by the ethics committees of both participating institutions, and written informed consent was obtained from all patients prior to inclusion in the study. Patients were identified from institutional operative logs and electronic medical records, and radiographic data were retrieved from the digital imaging archives. No formal a priori sample size calculation was performed because all eligible patients treated during the study period were included. We evaluated 123 consecutive patients who underwent surgery for OVF between January 2021 and July 2025 and were treated with either BKP (*n* = 99) or VBS (*n* = 24). OVF was diagnosed based on clinical symptoms combined with characteristic findings on lateral radiographs, MRI, and CT, including decreased vertebral height and signal changes consistent with acute or subacute fracture. Eligible patients had documented osteoporosis, defined as a bone mineral density (BMD) T-score of <−1.5 SD. Patients were excluded if they had severe pre-existing spinal deformity, underwent BKP or VBS at more than one vertebral level, had less than 6 months of follow-up, were unable to complete the VAS score, had a history of spinal tumor, or had severe physical illnesses such as Parkinson’s disease (Figure 1). Indications for surgical intervention included intractable back pain refractory to conservative management, established nonunion, or imaging features suggestive of a high risk of nonunion, such as high-intensity or diffuse low-intensity signal changes on T2-weighted MRI. In our institutions, specific indications for treatment with VBS were complex fracture patterns, including severe endplate injury, split-type fractures, and the absence of interbody sclerosis, whereas patients who did not meet these criteria were treated with BKP.

### 2.2. Clinical and Radiological Assessments

**Figure 2:**
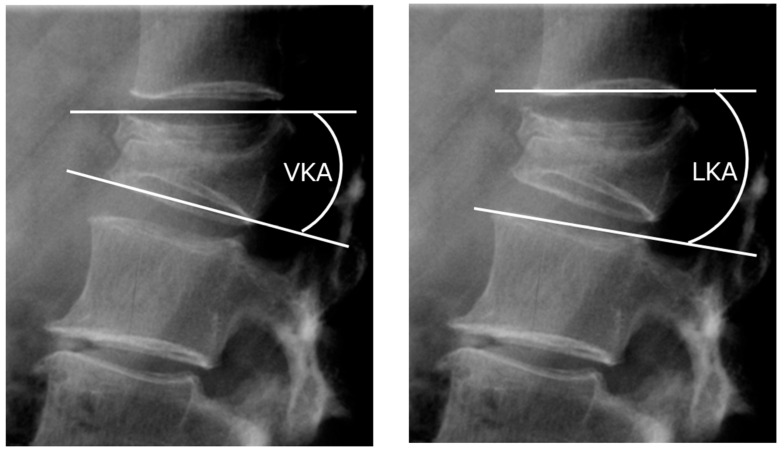
VKA and LKA, Vertebral kyphosis angle (VKA) is measured as the angle between the upper and lower end plates of the fractured vertebra. Local kyphosis angle (LKA) is measured as the angle between one vertebra above and one below it.

Patients underwent clinical assessment using the Visual Analogue Scale (VAS) preoperatively and at the first postoperative assessment. Clinical data, including BMD T-scores, vertebral fracture level, duration of surgery, occurrence of adjacent vertebral fracture, and need for revision surgery, were recorded during the perioperative period and on subsequent patient follow-up. Adjacent vertebral fractures were defined as new osteoporotic vertebral fractures occurring at one level above or below the treated vertebra during the follow-up period. For radiologic assessment, vertebral kyphosis angle (VKA) and local kyphosis angle (LKA) were measured on lateral radiographs before surgery, after surgery, and at final follow-up. VKA was measured as the angle between the superior and inferior endplates of the fractured vertebra, while LKA was the angle of kyphosis measured at the level above and below the fractured vertebra (Figure 2). Cement leakage was assessed on postoperative radiographs and, when clinically indicated, CT scans, and was defined as any extravasation of cement into the disc, epidural space, or paravertebral tissues.

### 2.3. Surgical Procedure

**Figure 3:**
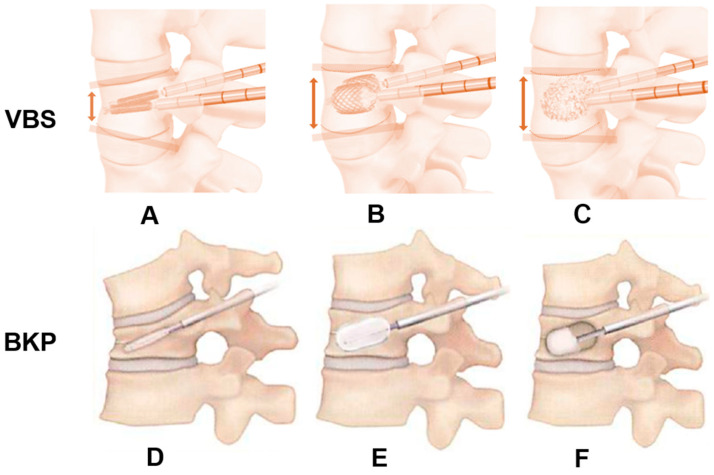
Surgical technique, Vertebral Body Stenting (VBS): (**A**): Stent insertion, (**B**): Enlargement of a stent, (**C**): Cementing, Balloon Kyphoplasty (BKP): (**D**): Balloon insertion, (**E**): Enlargement of a balloon, (**F**): Cementing. Red arrows and lines indicate that the anterior vertebral height is increased by the stent.

All surgeries were done under general anesthesia, with patients positioned prone on a radiolucent operating table. The technique by Lieberman [12] was adopted. Biplanar fluoroscopy was used to identify the target vertebral level, skin entry point, and pedicle trajectory. 1% lidocaine was infiltrated subcutaneously prior to skin incision. A transpedicular channel was established on each pedicle using a cannulated obturator over a guidewire. A working cannula was then placed over the obturator and advanced under fluoroscopic guidance (Figure 3).

For BKP, either the Kyphon™ balloon kyphoplasty platform, (Medtronic, Minneapolis, MN, USA) or the KMC Kyphoplasty System™ (Shanghai Kinetic Medical Co., Ltd., Shanghai, China) and Mendec Spine Bone Cement Kit™ (Nippon Americare Co., Ltd., Miami, FL, USA) was used. Balloon tamp was advanced into the vertebral body through the working cannula and gradually inflated under fluoroscopy (maximum pressure < 220 psi, approximately 4 mL of contrast medium per balloon) until vertebral height restoration or a plateau in expansion was observed. The balloons were then deflated and removed, and the resulting cavity was carefully filled with polymethylmethacrylate bone cement at low pressure under continuous fluoroscopic monitoring to minimize cement leakage. All instruments were removed after cement polymerization and hardening (Figure 3).

VBS was performed mainly for cases with endplate damage and split-type fractures. For VBS procedures, the Vertebral Body Stenting system™ (DePuy Synthes, Raynham, MA, USA) was utilized. Following creation of transpedicular access (Figure 3A), height restoration was initially achieved by inflating a vertebral body balloon under AP and lateral fluoroscopic control, after which an expandable VBS stent was placed in this prepared space (Figure 3B) and subsequently filled with polymethylmethacrylate cement as described in BKP (Figure 3C).

All procedures were performed by spine surgeons with more than 50 prior vertebral augmentation cases. Perioperative intravenous antibiotic prophylaxis and mechanical thromboprophylaxis were administered according to institutional protocols, and patients were mobilized out of bed on the first postoperative day as tolerated. Osteoporosis treatment (e.g., bisphosphonates, teriparatide, or romosozumab) was initiated in collaboration with the attending physicians in accordance with national guidelines.

### 2.4. Statistical Analysis

All data are presented as mean ± standard deviation (SD) for continuous variables and as counts and percentages for nominal or ordinal variables. Normality was assessed using the Shapiro–Wilk test. For continuous variables, the independent-samples *t*-test was used for normally distributed data, whereas the Mann–Whitney U test was used for non-normally distributed data. Categorical variables were analyzed using the Chi-squared test or Fisher’s exact test as appropriate. A *p*-value < 0.05 was considered statistically significant. All statistical analyses were performed using SPSS version 29.0.2.0 (20) (IBM, Beijing, China). All available data for eligible patients were included in the analyses, and cases with missing key variables were excluded from the corresponding analyses only. Because the study was retrospective, we analyzed available cases only and did not apply statistical methods to impute missing data.

## 3. Results

### 3.1. Patient and Surgical Factors

A total of 123 patients were included, with 24 treated with VBS and 99 with BKP. Baseline characteristics were comparable between groups—mean age was 80.6 ± 7.3 for the VBS groups and 80.8 ± 7.2 years for the BKP groups (*p* = 0.92), and sex distribution did not differ significantly (*p* = 0.25). Bone mineral density (BMD) T-scores were also similar (*p* = 0.74) (Table 1). Fracture level distribution was likewise comparable between the VBS and BKP groups (thoracic 29% vs. 36%, lumbar 71% vs. 64%, *p* = 0.34) (Table 1).

### 3.2. Clinical Result

Both VBS and BKP resulted in substantial and statistically significant reductions in back pain (*p* < 0.001), and postoperative VAS scores were similar between groups despite higher baseline pain in the VBS cohort. Preoperative VAS scores were higher in the VBS group (89 ± 18 vs. 73 ± 20; *p* = 0.03), and postoperative VAS scores were comparable between groups (18 ± 11 for VBS and 13 ± 12 for BKP; *p* = 0.06). The mean operative time was significantly longer in the VBS group than in the BKP group (39 ± 6 vs. 35 ± 9 min, *p* = 0.007). Cement volume infiltrated was similar between VBS and BKP (6.4 ± 1.4 vs. 6.7 ± 1.9 mL, *p* = 0.34). The incidence of subsequent adjacent vertebral fractures was also comparable (13% in VBS vs. 26% in BKP, *p* = 0.12). Additional surgery was required in 1 patient (4%) in the VBS group and in 8 patients (8%) in the BKP group, with no significant difference between groups (*p* = 0.44) (Table 2).

### 3.3. Radiological Result

**Figure 4:**
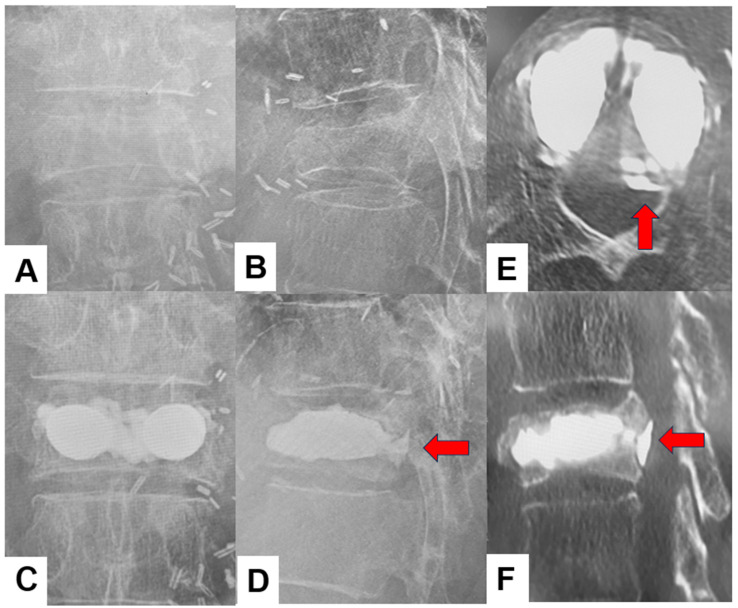
Cement leakage case of VBS, 84-year-old female of T12 OVF, (**A**): Preoperative anteroposterior radiogram, (**B**): Preoperative lateral radiogram, (**C**): Final follow-up anteroposterior radiogram, (**D**): Final follow-up lateral radiogram, (**E**): Axial CT, (**F**): Sagittal reconstruction CT. Red arrows indicate the cement leaked into the spinal canal.

VKA was comparable between groups preoperatively (13.6 ± 10.0° for VBS vs. 14.1 ± 7.1° for BKP; *p* = 0.39). Immediately after surgery, the VBS group had a significantly smaller VKA than the BKP group (4.8 ± 4.4° vs. 7.0 ± 4.9°; *p* = 0.03), indicating slightly better early kyphosis correction. However, the degree of VKA correction and the VKA at final follow-up were not significantly different between groups (VKA correction: 8.7 ± 7.1° vs. 7.2 ± 5.7°, *p* = 0.48; final follow-up: 7.8 ± 4.4° vs. 9.8 ± 6.0°, *p* = 0.23), suggesting that this initial radiographic advantage of VBS did not persist. LKA likewise showed no significant differences between the two groups at any time point (preoperative: 12.2 ± 8.9° vs. 9.0 ± 6.8°, *p* = 0.12; postoperative: 8.9 ± 9.5° vs. 6.6 ± 5.3°, *p* = 0.92; final follow-up: 10.6 ± 9.2° vs. 7.7 ± 5.9°, *p* = 0.32). Likewise, LKA correction was comparable between the two groups (1.2 ± 3.7° vs. 1.3 ± 6.0°, *p* = 0.41). Cement leakage, such as that shown in Figure 4, was significantly more frequent in the VBS group than in the BKP group (54% vs. 24%, *p* = 0.005) (Table 3). Cement leakage volume in both groups was very small and did not cause any clinical adverse effects.

## 4. Discussion

In our cohort, OVF patients treated with VBS had higher baseline VAS scores (89 ± 18 mm vs. 73 ± 20 mm, *p* = 0.03). However, both procedures achieved significant pain reduction (*p* < 0.001) and comparable postoperative VAS scores (18 ± 11 mm vs. 13 ± 12 mm, *p* = 0.06). In our practice, VBS was indicated for split-type fractures and severe endplate disruption. Since endplate deficits have been described as independent predictors of prolonged back pain after osteoporotic vertebral fractures [27], the higher baseline pain in the VBS group likely reflects the underlying fracture morphology. Regarding pain outcomes, previous reports have shown that BKP and VBS achieve similar pain relief, with comparable VAS or NRS scores [22,25,28,29]. Our findings are consistent with these studies and may further indicate a benefit, as the VBS group demonstrated comparable postoperative pain outcomes despite higher preoperative VAS scores and more complex fracture patterns.

Perioperatively, we found that the mean operative time was significantly longer in the VBS group than in the BKP group (39 ± 6 vs. 35 ± 9 min, *p* = 0.007). This is consistent with previous reports, in which BKP procedures typically required 22–35 min and VBS 35–42.5 min, with VBS consistently associated with longer operating times [25,26,28]. The longer procedure time with VBS may be multifactorial. Particularly in VBS, an additional step is balloon inflation to create a temporary cavity prior to actual stent deployment [29]. Additionally, the VBS device has a larger needle and a metal bracket, which may require the surgeon to be more careful and precise with catheter placement and balloon expansion [30].

Preoperative VKA was similar between VBS and BKP, and VBS achieved a smaller immediate postoperative VKA. However, the overall magnitude of correction did not differ between techniques. Preoperative and postoperative LKA, as well as the degree of correction, were likewise comparable. Disch et al. [19] demonstrated in a biomechanical model that, although BKP and VBS achieved similar initial kyphosis correction after balloon inflation, kyphosis loss after deflation was substantially greater with BKP (4.4° vs. 1.6°). Rotter et al. [21] similarly reported that both systems restore vertebral height under full inflation, but BKP shows a greater loss of reduction after deflation, with anterior height loss of 12% vs. 4% and a 58% vs. 21% loss of realignment height, resulting in a larger net anterior height gain with VBS (13% vs. 8%). Despite the favorable biomechanical data, clinical studies have not demonstrated clear radiographic superiority of VBS over BKP. In a randomized trial, Werner et al. [24] reported mean kyphosis reductions of 4.5 ± 3.6° with BKP and 4.7 ± 4.2° with VBS, with no significant difference between the two groups. Yokoyama et al. [22] found comparable gains in vertebral height and local kyphosis angle (6.9 ± 7.0° for BKP vs. 5.4 ± 3.4° for VBS; *p* = 0.51), and Vendeuvre et al. [23] likewise noted significant improvements in kyphosis and anterior vertebral height in both groups without any significant intergroup difference at postoperative assessment.

At final follow-up, both VKA and LKA were similar between groups, suggesting that the initial postoperative advantage of VBS in VKA did not persist over time and may reflect minor secondary sintering and loss of kyphosis correction, as previously described by Hartmann et al. [31]. This is in line with the findings of Enyo et al. [26], who reported no significant differences at 3 and 6 months, and Maeda et al. [25], who found no significant differences at 1 year in anterior and posterior vertebral body height, wedge angle, or local kyphotic angle. Importantly, we observed these equivalent radiographic outcomes despite the preferential use of VBS in more complex fracture patterns characterized by split-type morphology and endplate disruption. 

A cement volume above 4.5 mL has previously been suggested as sufficient to obtain pain relief [32], and the mean volumes in both of our groups (6.4 ± 1.4 vs. 6.7 ± 1.9 mL, *p* = 0.34) exceed this threshold. In our cohort, however, cement leakage was significantly more frequent in the VBS group than in the BKP group (54% vs. 24%; *p* = 0.005) (Figure 4). Reported cement leakage rates in the literature vary widely, ranging from 8.6–41.7% for BKP [11,13,22,23,24,28,33,34] and 4.2–44% for VBS [11,24,28,33]. Vendeuvre et al. [23] previously reported a markedly lower leakage rate with VBS than with BKP (41.7%vs. 4.2%) in patients with non-osteoporotic thoracolumbar fractures. More recent studies focusing on OVF, however, have not demonstrated a consistent difference in leakage incidence between BKP and VBS [24,26,28]. The increased incidence of leakage in the VBS group may primarily reflect our selection of VBS for cases with endplate damage and split-type fractures, as disruption of cortical bone integrity has been identified as an important predictor of cement leakage [35].

In our study, the incidence of adjacent vertebral fractures was similar between groups (13% in the VBS group vs. 26% in the BKP group, *p* = 0.12). Reported rates of adjacent vertebral fractures in the literature vary widely, ranging from 6.8–21.0% for VBS [25,26,33,36] and 3.52–28.6% in BKP [18,25,26,37,38]. Our findings are consistent with previous studies comparing the two groups, which found no difference in the incidence of subsequent adjacent vertebral fractures [25,26]. These data suggest that an adjacent vertebral fracture may be influenced by other previously identified factors, such as low bone mineral density [39,40,41], advanced age [41], reduced local kyphosis [39], and intradiscal cement leakage [40,41,42]. Similarly, the need for additional surgery was not significantly different between groups (4% vs. 8%, *p* = 0.44). Notably, in our clinical practice, VBS was indicated for fractures with endplate injury and split-type morphology, which have been identified as high-risk patterns for revision after BKP. Takahashi et al. reported that these fracture types were strongly associated with reoperation after BKP alone and instead suggested that BKP be combined with short-segment instrumentation as an adjunct [43]. In addition, cement dislodgement has previously been seen in split-type fractures. In response to this risk, Yonezawa et al. [44] proposed vertebra-pediculoplasty with cement injection via a cannulated screw for these complex fracture morphologies. In this context, the comparable revision rates and adjacent fracture rates observed between the VBS and BKP groups may indicate a relative benefit for VBS, achieving similar outcomes despite the more complex fracture morphology in this cohort. However, this interpretation should be made with caution, given the study design.

Our study has several practical implications for the management of OVF. The comparable pain relief and radiographic alignment achieved with VBS and BKP suggest that BKP remains an appropriate option for uncomplicated osteoporotic fractures. Furthermore, the similar mid-term outcomes observed in VBS despite more complex fracture morphology support its use as a feasible alternative for split-type fractures and severe endplate defects, in which BKP alone may be associated with a higher risk of failure or reoperation.

This study has several limitations. First, there is an inherent indication bias, as severe endplate injuries and split-type fractures were treated with VBS, resulting in differences in fracture complexity between the two groups. Second, the VBS cohort was relatively small, limiting statistical power to detect modest differences in outcomes. Finally, with a short follow-up period, late complications may be underestimated. These factors should be considered when interpreting our results, and further prospective studies with a longer follow-up period are warranted.

Future research should include adequately powered prospective or randomized studies that stratify patients according to fracture morphology and endplate integrity to clarify which subgroups benefit most from VBS versus BKP. Incorporating patient-reported outcomes, health-related quality of life, and cost-effectiveness analyses will also be important to inform treatment decisions in aging populations. Additionally, imaging-based studies focusing on predictors of cement leakage and adjacent fractures, specifically after VBS, could help refine patient selection and procedural techniques.

## 5. Conclusions

In this multicenter cohort, VBS and BKP provided comparable improvements in pain, radiographic alignment, and mid-term outcomes, including adjacent vertebral fractures and revision surgery rates. These findings suggest that VBS is a viable option for OVF with split-type morphology and severe endplate damage, achieving similar outcomes comparable to BKP despite greater fracture complexity. However, a clear advantage of VBS over BKP has not been demonstrated, and prospective studies with longer follow-up and stratification by fracture morphology are necessary to determine whether VBS is superior to BKP.

## Figures and Tables

**Table 1:** Comparison of patient demographics and characteristics between the VBS and BKP groups.

Variable	VBS *n* = 24	BKP *n* = 99	*p*-Value
Age (years)	80.6 ± 7.3	80.8 ± 7.2	0.92
Sex (Male/Female)	12/12	36/63	0.25
T-score	−2.1 ± 1.2	−2.0 ± 1.3	0.74
Fracture Level			0.34
Thoracic	7 (29%)	36 (36%)	
Lumbar	17 (71%)	63 (64%)	

Values expressed as mean ± SD or counts (percentage).

**Table 2:** Comparison of operative data and clinical outcomes.

Variable	VBS *n* = 24	BKP *n* = 99	*p*-Value	Cohen’s d	95% CI
VAS (mm)					
Preoperative	89 ± 18	73 ± 20	0.03 *	0.84	73.1–80.1
Postoperative	18 ± 11	13 ± 12	0.06	0.41	12.5–16.7
Post-Preoperative	63 ± 17	60 ± 22	0.26	0.15	57.3–64.7
Operative time (mins)	39 ± 6	35 ± 9	0.007 *	0.46	31.0–37.1
Cement volume (mL)	6.4 ± 1.4	6.7 ± 1.9	0.34	0.18	6.34–6.97
Adjacent vertebral fracture	3 (13%)	26 (26%)	0.12		
Additional surgery	1 (4.2%)	8 (8.1%)	0.44		

Values expressed as mean ± SD or percentage, * statistically significant difference *p*-value < 0.05, 95% CI: 95% confidential interval.

**Table 3:** Comparison of radiographic findings between the VBS and BKP groups.

Variable	VBS *n* = 24	BKP *n* = 99	*p*-Value	Cohen’s d	95% CI
Preoperative	13.6 ± 10.0°	14.1 ± 7.1°	0.39	0.06	12.7–15.4
Postoperative	4.8 ± 4.4°	7.0 ± 4.9°	0.03 *	0.46	5.68–7.38
Correction	8.7 ± 7.1°	7.2 ± 5.7°	0.48	0.25	6.48–8.58
Final follow-up	7.8 ± 4.4°	9.8 ± 6.0°	0.23	0.34	8.37–10.4
*Local Kyphosis Angle*					
Preoperative	12.2 ± 8.9°	9.0 ± 6.8°	0.12	0.44	8.31–10.9
Postoperative	8.9 ± 9.5°	6.6 ± 5.3°	0.92	0.35	5.91–8.16
Correction	1.2 ± 3.7°	1.3 ± 6.0°	0.41	0.02	1.33–3.92
Final follow-up	10.6 ± 9.2°	7.7 ± 5.9°	0.32	0.50	7.10–9.44
Cement Leakage	13 (54%)	24 (24%)	0.005 **		

Values expressed as mean ± SD or percentage, * statistically significant difference *p*-value < 0.05. ** statistically significant difference *p*-value < 0.01, 95% CI: 95% confidential interval.

## Data Availability

The data presented in this study are available in the article.
